# 5-HT_7_ receptor stimulation and blockade: a therapeutic paradox about memory formation and amnesia

**DOI:** 10.3389/fnbeh.2014.00207

**Published:** 2014-06-12

**Authors:** Alfredo Meneses

**Affiliations:** Departamento de Farmacobiología, Centro de Investigación y de Estudios Avanzados, Instituto Politécnico NacionalMexico City, Mexico

**Keywords:** 5-HT_7_ receptor, memory, dysfunctional memory, autoshaping, rats

## Overview

Mammalian memory involves multiple brain areas and neurotransmitter systems; both at the receptor and post-receptor level (Meneses, 2014a,b). Investigations of serotonin (5-hydroxytryptamine, 5-HT) involvement in memory (Meneses, 2013) have been significantly enhanced by the identification, classification, and cloning of multiple receptors (Hoyer et al., 1994, 2002) and studies at the post-receptor level (Raymond et al., 2001). Emergent investigation of 5-HT_7_ receptor is growing but contradictory. Hence, major current questions include (1) the paradox that 5-HT_7_ receptor agonists and antagonists in behavioral memory tasks are revealing promnesic and antiamnesic effects; (2) 5-HT_7_ and 5-HT_1A_ receptors interaction (and other 5-HT receptors), and neural markers associated to these cognitive processes and individual differences. In this work recent data are briefly revised.

## 5-HT_7_ receptor stimulation

Freret et al. (2014) reported that with a 2-h delay, (post-training) administration of the 5-HT_7_ receptor antagonist SB-269970 (3 and 10 mg/kg, sc) impaired the discrimination of the novel object; but with a 4-h delay, while control mice were not able to discriminate the novel object, mice treated with the agonist 5-carboxamidotryptamine (5-CT; displaying affinity for several receptors including 5-HT_7_; Hoyer et al., 1994) showed a significant discrimination. This promnesic effect was blocked by SB-269970, but not by WAY-100135 or GR-127935 (5-HT_1A_ or 5-HT_1B/1D_ receptor antagonists, respectively; Hoyer et al., 1994). Freret et al. (2014) conclude that 5-HT_7_ receptor tonically modulates cognitive processes involved in consolidation performances in object recognition and therefore, it could be a promising target to treat memory dysfunctions (especially episodically related deficits) or pathological aging. Notably, Stahl (2010) suggests that 5-HT_7_ receptor (blockade) as a novel therapeutic target for antidepressant and pro-cognitive effects. Nikiforuk et al. (2013) also conclude that antagonism of 5-HT_7_ receptor may represent a useful pharmacological approach in the treatment of cognitive deficits and some negative schizophrenia symptoms. Also, Tajiri et al. (2012) and Gasbarri and Pompili (2014) propose that the 5-HT_7_ receptor is a rational target for the treatment of psychiatric disorders. Certainly, more research is necessary about 5-HT_7_ receptor, its functional complexity in memory formation and abnormal memory as well timing of drug administration (McGaugh, 1989; Monleón et al., 2008).

Pre-training elevation of 5-HT by the selective serotonin reuptake inhibitor (SSRI) fluoxetine had no effect by itself, but facilitated passive avoidance when combined with the 5-HT_1A_ receptor antagonist NAD-299 and this facilitation was blocked by SB-269970 (Eriksson et al., 2012). Likewise Eriksson et al. (2012) reported that a reduced activation of the 5-HT_1A_ receptor was resulting in enhanced stimulation of the 5-HT_7_ receptor but 5-HT_7_ receptor agonists LP-44 or AS19 failed to facilitate passive avoidance performance; and according with these authors LP-44 and AS19 have low efficacy to stimulate protein phosphorylation of 5-HT_7_ receptor-activated signaling cascades. Notably, in the mutant mouse brain (lacking pituitary adenylate cyclase, an experimental mouse model for psychiatric disorders) 5-HT_7_ protein expression did not differ from wild-type mice but in primary embryonic hippocampal neurons AS-19 increased neurite length and number (Tajiri et al., 2012). Likewise, LP-211 and 8-OHDPAT affect neurites in embryonic cultures (Speranza et al., 2013). Certainly, Eriksson et al. (2012) propose that retention (in passive avoidance) is mediated through hippocampal 5-HT_1A_ receptor activation, while the 5-HT_7_ receptor appears to facilitate memory processes in a broader cortico-limbic network and not the hippocampus alone. Notably, LP-211 rescued diverse defective performances, including memory in the novelty preference task as well as the abnormal activation of PAK and cofilin (key regulators of actin cytoskeleton dynamics) and of the ribosomal protein (rp) S6, whose reduced activation in MECP2 mutant neurons is responsible for the altered protein translational control (De Filippis et al., 2014). De Filippis et al. (2014) indicate that pharmacological targeting of 5-HT_7_ receptor improves specific behavioral and molecular manifestations and these data are a first step toward the validation of an innovative systemic treatment to disorders associated with intellectual disability.

Importantly, either AS-19 or LP-211 or the dual 5-HT_1A/7_ receptor agonist, 8-OHDPAT, facilitated memory consolidation in an associative memory task and increased cAMP production (Pérez-García et al., 2006; Meneses, 2013; Meneses et al., 2014) which were reversed by SB-269970. Certainly, an association between memory and cAMP production exists (Kandel, 2001; Izquierdo et al., 2006). It should be noted however that memory formation in an autoshaping learning (Pérez-García and Meneses, 2008) showed that cAMP production was decreased by 8-OHDPAT if memory was improving but the opposite occurred in absence of memory. In autoshaping task, trained animals are food-restricted (at 85% of *ad-libitum)*, receiving one autoshaping training and are tested at 1.5 h for short-term memory (STM) and at 24 and 48 h for consolidation of long-term memory (LTM) (Meneses, 2013). Why 8-OHDPAT improved memory (consolidation, 48 h) and increased cAMP in cortex and hippocampus (Manuel-Apolinar and Meneses, 2004) or decremented (Pérez-García and Meneses, 2008) hippocampal cAMP production? Importantly, the new 5-HT_7_ receptor agonist LP-211 did not affect STM; nonetheless, at 0.5 and 1.0 mg/kg it improved LTM. The 5-HT_7_ receptor antagonist SB-269970 (10.0 mg/kg) alone had not effect but it reversed the LP-211 (1.0 mg/kg) LTM facilitation. The scopolamine (0.2 mg/kg) induced-decrement in CR was accompanied by significant increased cAMP production. Scopolamine-induced amnesia and increments in cAMP, are significantly but not completely reversed by LP-211 (Meneses et al., 2014). Hence, prefrontal cortex, cAMP production and improved memory formation seem to be associated; however, this association is complex and dependent on the basal level (e.g., prevalent expression of either of 5-HT_1A_ or 5-HT_7_ receptors). For instance, the time-course (0–120 h) of autoshaped responses revealed progressive performance and mRNA 5-HT_1A_ or 5-HT_7_ receptors are monotonically augmented or declined in prefrontal cortex, hippocampus and raphe nuclei, respectively (Perez-Garcia and Meneses, 2009). Moreover, 5-HT_1A_ receptor is increased, whereas 5-HT_7_ receptor levels are decreased by aging (Saroja et al., 2014). Also, they showed significant correlation with the time spent in target quadrant; hence according with Saroja et al. (2014) these are two key parameter of memory retrieval which in turn unambiguously links the serotonergic receptor system to spatial memory performance.

## 5-HT_7_ receptor blockade: cognitive demand or memory impaired

Gasbarri et al. (2008) showed that SB-269970 improved memory, decreasing the number of errors in test phase and, thus, affecting reference memory, while no effects in working memory; postulating that 5-HT_7_ receptor blockade had procognitive effect, when the learning task implicated a high degree of difficulty. In addition, the 5-HT_7_ receptor antagonists, SB-269970 or DR 4004 alone had no effect but reversed amnesia induced by scopolamine and dizocilpine (Meneses, 2004). Hence, 5-HT_7_ receptor antagonism plays an important role under poor memory or when the learning or memory is complex.

## 5-HT_7_ blockade and promiscuous affinity

In recent time, it has become evident that 5-HT_7_ blockade and drugs displaying promiscuous affinity have interesting effects. For instance, lurasidone (affinity for several receptors including 5-HT_7_) and the selective 5-HT_7_ receptor antagonist, SB-656104-A improved learning and memory deficits by dizocilpine (or MK-801), in the rat passive avoidance test, and AS-19 (3 mg/kg) completely blocked the attenuating effects of lurasidone (3 mg/kg); AS-19 (1–10 mg/kg) pre-training administration had no effects (Horisawa et al., 2013). SB-269970 (30 but not 10 mg/kg) pre-training administration produces both anti-psychotic-like (amphetamine- or phencyclidine-induced hyperactivity tests) and pro-cognitive (novel object discrimination test) activity in preclinical animal models (Waters et al., 2012); these authors conclude that SB-269970 is more a potent inverse agonist than SB-258741, which might be a potential explanation for the conflicting profiles *in vivo* (for references see Waters et al., 2012). Importantly, Huang et al. (2014) reported that 5-HT_1A_ and 5-HT_7_ receptors contribute to lurasidone-induced dopamine efflux, concluding that, at least partially, 5-HT_1A_ agonist and 5-HT_7_ antagonist properties may contribute to reversing schizophrenia-like effects. Chronic stress impaired performance on the extra-dimensional (ED) set-shifting stage of the frontal-dependent attentional set-shifting task and amisulpride (3 mg/kg) before testing reversed this restraint-induced cognitive inflexibility and improved ED performance of the unstressed control group (Nikiforuk and Popik, 2013). AS-19 (10 mg/kg) pre-training alone had no effect but abolished the pro-cognitive efficacy of amisulpride (Nikiforuk and Popik, 2013). Nikiforuk et al. (2013) also reported that acute administration of SB-269970 (1 mg/kg) or amisulpride (3 mg/kg) ameliorated ketamine-induced cognitive inflexibility and novel object recognition deficit in rats; both compounds were also effective in attenuating ketamine-evoked disruption of social interactions. In contrast, neither SB-269970 nor amisulpride affected ketamine-disrupted prepulse inhibition; ketamine is a glutamatergic antagonist (Neill et al., 2010). Importantly, in contrast to the negative regulatory effects of long-term activation of 5-HT_7_ receptors on NMDA receptor signaling (*in vitro*) acute activation of 5-HT_7_ receptors promotes NMDA receptor activity (Vasefi et al., 2013). Hence, these findings highlight the potential for temporally differential regulation of NMDA receptors by the 5-HT_7_ receptor. While some inconsistencies might be related to the opposite action exerted by 5-HT_1A_ and 5-HT_7_ receptors over cAMP production (Renner et al., 2012; Meneses, 2014a,b); certainly, in different protocols of training/testing (memory consolidation vs. STM and LTM), various brain areas and neurotransmission systems interaction might be also implicated.

## 5-HT_1a_ and 5-ht_7_ receptors

The functional significance of 5-HT_1A_ and 5-HT_7_ receptors dimerization is has been revised (Matthys et al., 2011; Gellynck et al., 2013; Herrick-Davis, 2013); indicating that it differentially regulates receptor signaling and trafficking (Renner et al., 2012). But still we do not know the implications of this in memory formation and amnesic conditions; hence, the study of signaling associated to 5-HT_7_ receptor in memory formation, amnesia and forgetting might provide significant insights (Meneses, 2014a,b). For instance, while the association of 5-HT_7_ receptor stimulation, improved memory and increased cAMP seems to be reliable findings; certainly factors such as differential regulation of hippocampal expression as well as individual differences might be important (see Meneses, 2013). In addition, a biphasic and brain-region selective down-regulation of cAMP concentrations is observed supporting object recognition in the rat (Hotte et al., 2012). Wang et al. (2013) have noted that while it is relatively well established that cAMP signaling is involved in the mediation of memory, the reports on its role to date are inconsistent. One hypothesis is that overactive cAMP signaling impairs working memory in the aged prefrontal cortex (PFC) or activation of the cAMP signaling in the frontal cortex is necessary for working memory; the explanations for this discrepancy may include: (i) activation of cAMP signaling within the PFC and an inverted U-shape dose-response on working memory and memory optimum range of cAMP rather than an overmuch or scanty production; (ii) the continuous and dynamic updating of cAMP levels occurs at the different time-course of memory formation; (iii) cAMP activation might be beneficial for working memory under conditions that require hippocampal–PFC interactions (Wang et al., 2013). Given the complexity of cAMP-dependent responses; hence studies of brain areas and individual differences remain to be reported at both behavioral and cellular levels (see Wang et al., 2013).

Evidence exists of individual differences regarding memory (Ballaz et al., 2007a,b; Fitzpatrick et al., 2013; Flagel et al., 2014) and forgetting (Tellez et al., 2012); hence, it seems reasonable to suggest that in autoshaping (or sign-tracking; see Meneses, 2003) the increment (Manuel-Apolinar and Meneses, 2004) or decrement (Pérez-García and Meneses, 2008) cAMP production might be involving individual differences (e.g., Meneses, 2014a,b). Notably, individual variation in the magnitude and influence of cue reactivity over behavior in humans and animals suggest that cue-reactive individuals may be at greater risk for the progression to addiction and/or relapse (Anastasio et al., 2014).

Also, an important implication is that 5-HT_7_ (and/or 5-HT_1A_) receptors stimulation increased or decreased cAMP production (e.g., Hoyer et al., 1994) and improved memory (Meneses et al., 2014). Likewise, evidence indicates that 5-HT_7_ splice variants constitutively activate adenylyl cyclase (Leopoldo et al., 2011). It should be crucial to confirm if memory formation, amnesia, or forgetting by themselves and/or plus drugs modify adenylyl cyclase.

Hence, (1) memory requires restricted or selective cAMP production (Pérez-García and Meneses, 2008; Meneses, 2013); (2) a major and consistent emerging finding is that 5-HT_7_ receptor stimulation seems to facilitate memory formation and reverse memory impairment; (3) expression of 5-HT_7_ (and 5-HT_1A_) receptors are accompanying memory; (4) the 5-HT_7_ antagonism alone had no effect but reversed memory deficits. Notably, a combination of neural and cognitive processes may contribute an early and specific marker of disorders associated to dysfunctional cognitive skills or memory in psychiatric disorders (Millan et al., 2012), including Alzheimer's disease progression (Ibanez and Parra, 2014).

## Conclusions

Behavioral and molecular studies may be particularly insightful and timely in view of the apparently contradictory notion that either 5-HT_1A_ or 5-HT_7_ receptor agonists or antagonists are useful in the treatment of learning and memory disorders. Also, the distinction about normal memory or impaired memory, timing of drug administration and individual differences are providing important insights about 5-HT_1A_ or 5-HT_7_ receptors stimulation and blockade.

### Conflict of interest statement

The author declares that the research was conducted in the absence of any commercial or financial relationships that could be construed as a potential conflict of interest.
